# Raw and Fermented Alfalfa Brown Juice Induces Changes in the Germination and Development of French Marigold (*Tagetes patula* L.) Plants

**DOI:** 10.3390/plants10061076

**Published:** 2021-05-27

**Authors:** Döme Barna, Szilvia Kisvarga, Szilvia Kovács, Gábor Csatári, Ibolya O. Tóth, Miklós Gábor Fári, Tarek Alshaal, Nóra Bákonyi

**Affiliations:** 1 Department of Applied Plant Biology, Institute of Crop Sciences, University of Debrecen, Böszörményi Street 138, 4032 Debrecen, Hungary; szkovacs@agr.unideb.hu (S.K.); csatari.gabor@agr.unideb.hu (G.C.); olahne@agr.unideb.hu (I.O.T.); fari@agr.unideb.hu (M.G.F.); alshaaltarek@gmail.com (T.A.); nbakonyi@agr.unideb.hu (N.B.); 2 Research Institute for Fruit Growing and Ornamentals, National Agricultural Research and Innovation Center (NARIC) Szent-Györgyi Street 4, 2100 Gödöllő, Hungary; fullerina@gmail.com; 3 Soil and Water Department, Faculty of Agriculture, Kafrelsheikh University, Kafr El-Sheikh 33516, Egypt

**Keywords:** organic fertilizers, bioferilizers, biorefinery, alfalfa brown juice, lacto-fermentation, biostimulant, seed germination, ornamental plants, physiology, anatomy

## Abstract

Organic and ecological farming programs require new and efficient biostimulants with beneficial properties for the sustainable and safe production of seedlings and ornamental plants. We examined the effect of non-fermented and lacto-fermented alfalfa brown juice (BJ) on seed germination and the vegetative, physiological, and anatomical properties of French marigold (*Tagetes patula* L. ’Csemő’) plants which were treated with 0.5–10% fermented and non-fermented BJ, with tap water applied as a control. Applying 0.5% fermented BJ significantly improved seed germination compared with non-fermented BJ, resulting in an increase of 9.6, 11.2, 10.9, and 41.7% in the final germination percent, germination rate index, germination index, and vigor index, respectively. In addition, it increased the root and shoot length by 7.9 and 16.1%, respectively, root and shoot dry mass by 20 and 47.6%, respectively, and the number of leaves by 28.8% compared to the control. Furthermore, an increase in contents of water-soluble phenol, chlorophyll a and b, and carotenoid was reported upon the application of 0.5% fermented BJ, while peroxidase activity decreased. Our results prove that alfalfa BJ can be enrolled as a biostimulant as part of the circular farming approach which supports the sustainable horticultural practice.

## 1. Introduction

The ornamental plant industry is one of the most differentiated, multifaceted, and dynamic agricultural production sectors, and it is attracting growing attention from marketers and industrial stakeholders. However, ornamental plant companies should prioritize the use of environmentally friendly farming techniques, such as applying row materials with a low environmental impact to meet the criteria for the sustainable agriculture and the circular economy [1]. A recently-developed and novel technology enables the production of large amounts of an environmentally-friendly alfalfa-based organic biostimulant called brown juice (BJ), which is a by-product of leaf protein concentrate (LPC) [2]. The pH of fresh BJ varies between 5 and6, and it contains several biologically-active components, including phenols, amino acids, macro- and microelements, carbohydrates (40%), and nitrogen (3%) on a dry mass basis [3,4,5]. Due to its high sugar content, fresh BJ spoils within a week of storage at room temperature. Lacto-fermentation of BJ was found to increase its stability at room temperature, and significantly improve the nutritional characteristics of BJ due to the conversion of sugars into organic acids, which drop its pH down to approximately 4. After lacto-fermentation, BJ becomes more stable and effective as a value-added material for organic fertilization of horticultural and agricultural plants. Although the microbiome of fermented BJ is unknown, plant growth-promoting bacteria could provide an additional benefit [6,7] to the fermented BJ, which is an effective foliar biostimulant for plumed cockscomb and sweet basil [3,8].

The French marigold, *Tagetes patula* L., is native to the southwest of the United States, South Central Mexico, and Argentina. Many varieties are well known in horticulture, and there are records of its cultivation by Native American tribes [9]. French marigold plants are grown as ornamental and industrial plants under diverse climatic conditions [10], and several of their uses, characteristics, and properties have been studied. Research on French marigolds has included the investigation of essential oil production [11], antifungal properties such as their inhibitory effect on *Botrytis cinerea* and *Penicillium digitatum* [12], nectar [13], the larvicidal effect of thiophene in oil [14,15], cadmium toxicity suppression [16], lutein esters [17], karyotypes [18], and growth and flowering [19]. The effect of biofertilization on the seed germination and development of French marigolds is less well studied. The number of days French marigold requires for seed germination ranges from 10 to 14 days at temperatures between 21 and 22 °C. The effects of different organic manures on the seed germination of French marigolds were tested by Chander et al. [20], who noted that poultry manure positively stimulated the seed germination of French marigolds. To our knowledge, no reports have discussed the effect of plant-based biostimulants on seed germination in French marigolds, and limited studies have documented the effects of biofertilizers on plant development. Draghia et al. [21] and Raja [22] examined the effect of biofertilization on the growth and anatomical changes of French marigolds. Additionally, the effects of bio-pharmaceutical waste on French marigold plants have been tested in a pot experiment [23]. Mostly, slight but positive effects have been found. For example, the application of the biostimulant Radifarm^®^ enhances the growth traits, such as dry mass, number of leaves, nutritional status, flowering, and the development of shoot and root of French marigold plants [24], and it improves the uptake of nitrogen and potassium [25,26,27].

This study aimed to examine the influence of alfalfa BJ, before and after lacto-fermentation, on the seed germination, plant biometrics, and physiological and anatomical characteristics of French marigold plants. Our findings inform the use of alfalfa BJ as an environmentally friendly biostimulant that can be used in horticulture.

## 2. Results

### 2.1. Effect of Non-Fermented and Fermented BJ on Seed Germination

**Figure 1:**
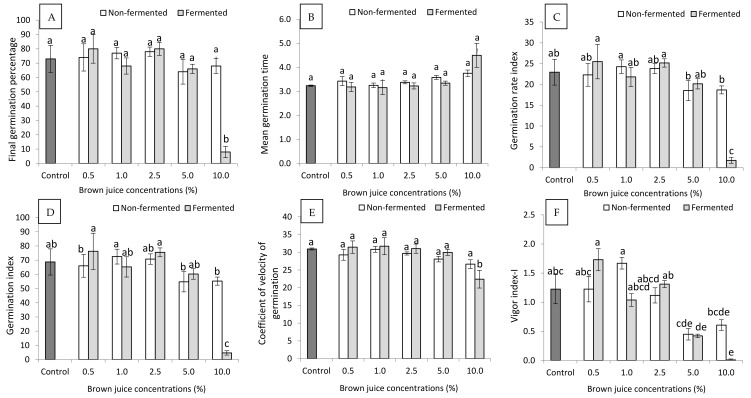
Effects of non-fermented and fermented alfalfa brown juice (BJ) on the (**A**) final germination percentage, (**B**) mean germination time, (**C**) germination rate index, (**D**) germination index, (**E**) coefficient of velocity of germination, and (**F**) vigor index-I of French marigold plants (*n* = 4). Data are presented as the mean ± standard deviation. Different letters on the top of the bars indicate significant differences (*p* ≤ 0.05).

The results of the seed germination experiment are presented in Figure 1. According to the statistical analysis, there were no significant differences between BJ treatments in the case of the FGP (1A) and CVG (1E) at *p* ≤ 0.05. Compared to the control treatment, the germination indices showed that the exogenous application of alfalfa BJ, either before or after lacto-fermentation, induced seed germination, particularly at the low concentrations of BJ up to 2.5%. Interestingly, higher concentrations (above 2.5%) of fermented and non-fermented BJ reduced the power of the germination process of the French marigold seeds. Treating seeds with 0.5% of fermented BJ increased the FGP from 73% (control) to 80%, while the same concentration of non-fermented BJ showed a FGP of 74%. Additionally, the application of 2.5% of fermented and non-fermented BJ resulted in FGPs of 80% and 78%, respectively. Higher concentrations of 5% and 10% exhibited lower FGPs than the control for both fermented and non-fermented BJ (Figure 1A). The control seeds had an MGT of 3.2 days, and none of the treatments of BJ, including fermented and non-fermented BJ, showed a lower MGT than the control treatment. However, the treatments with 0.5%, 1.0%, and 2.5% of fermented BJ exhibited the same MGT as the control treatment. Higher rates of fermented BJ, i.e., 5.0% and 10.0%, had MGTs of 3.3 and 4.5 days, respectively. Contrarily, the plants treated with non-fermented BJ exhibited higher MGTs than the control treatment, and increasing the concentration of non-fermented BJ resulted in a higher MGT (Figure 1B). The plants which received low concentrations of non-fermented (i.e., 1.0% and 2.5%) and fermented (i.e., 0.5% and 2.5%) BJ had higher GRIs than the control plants. Moreover, the fermented BJ displayed a higher synergetic effect on GRI than the non-fermented BJ; applying non-fermented and fermented BJ at the rate of 2.5% recorded GRIs of 23.8 and 25.2, respectively. However, the highest GRI (25.5) corresponded to seeds which received 0.5% of the fermented BJ, while the control plants had a GRI of 23.0 (Figure 1C). The GI results were consistent with those reported above for GRI. The highest GI (76.3) was measured in seeds treated with 0.5% of the fermented BJ, followed by the treatment of 2.5% of fermented BJ, which had a GI of 75.5. Plants in the fermented BJ treatments had higher GI values than those in the non-fermented treatments, except when BJ was applied at concentrations of 1.0% and 10.0%. The control plants had a GI of 68.8, which is lower than the GIs measured for plants treated with 1.0% and 2.5% of non-fermented BJ and 0.5% and 2.5% of fermented BJ (Figure 1D). Only seeds treated with fermented BJ at concentrations of 0.5%, 1.0%, and 2.5% had higher CVG than the control. Other treatments, especially the non-fermented BJ, exhibited lower CVG than the control treatment. The highest CVG (31.7) corresponded to the treatment of 1.0% fermented BJ, while germinated seeds in the control treatment showed a CVG of 30.9. For the non-fermented BJ treatments, the highest CVG (30.8) was observed for the seeds germinated in the presence of 1.0% of BJ. However, higher doses of non-fermented BJ linearly reduced the CVG. Similarly, concentration of fermented BJ above 1.0% resulted in a gradual decrease in the CVG (Figure 1E). Notably, fermented BJ displayed better effects on seed germination than non-fermented BJ, particularly at low concentrations. At high concentrations, i.e., 5.0% and 10.0%, higher values of VI-I were measured for germinated seeds treated with non-fermented BJ than those with fermented BJ. However, these values were below 50% lower than control. The highest VI-I (1.7) was measured for the treatments of 0.5% fermented BJ and 1.0% non- fermented BJ, and this value was 42% higher than the control. Additionally, the treatment of 2.5% fermented BJ showed a higher VI-I value than the control (Figure 1F).

### 2.2. Development of French Marigold Plants Treated with BJ

#### 2.2.1. Plant Biometric Parameters

**Figure 2:**
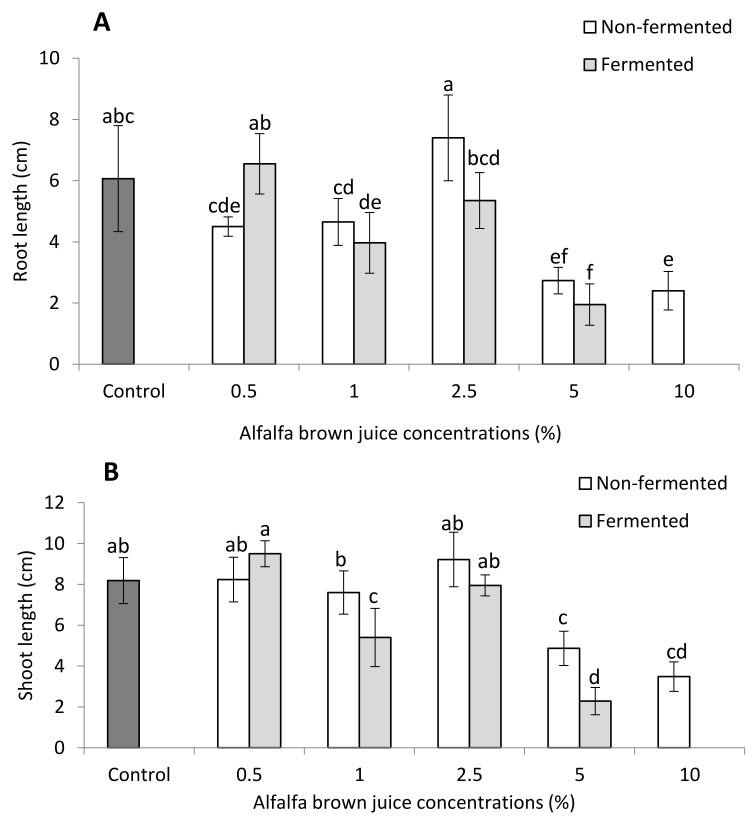

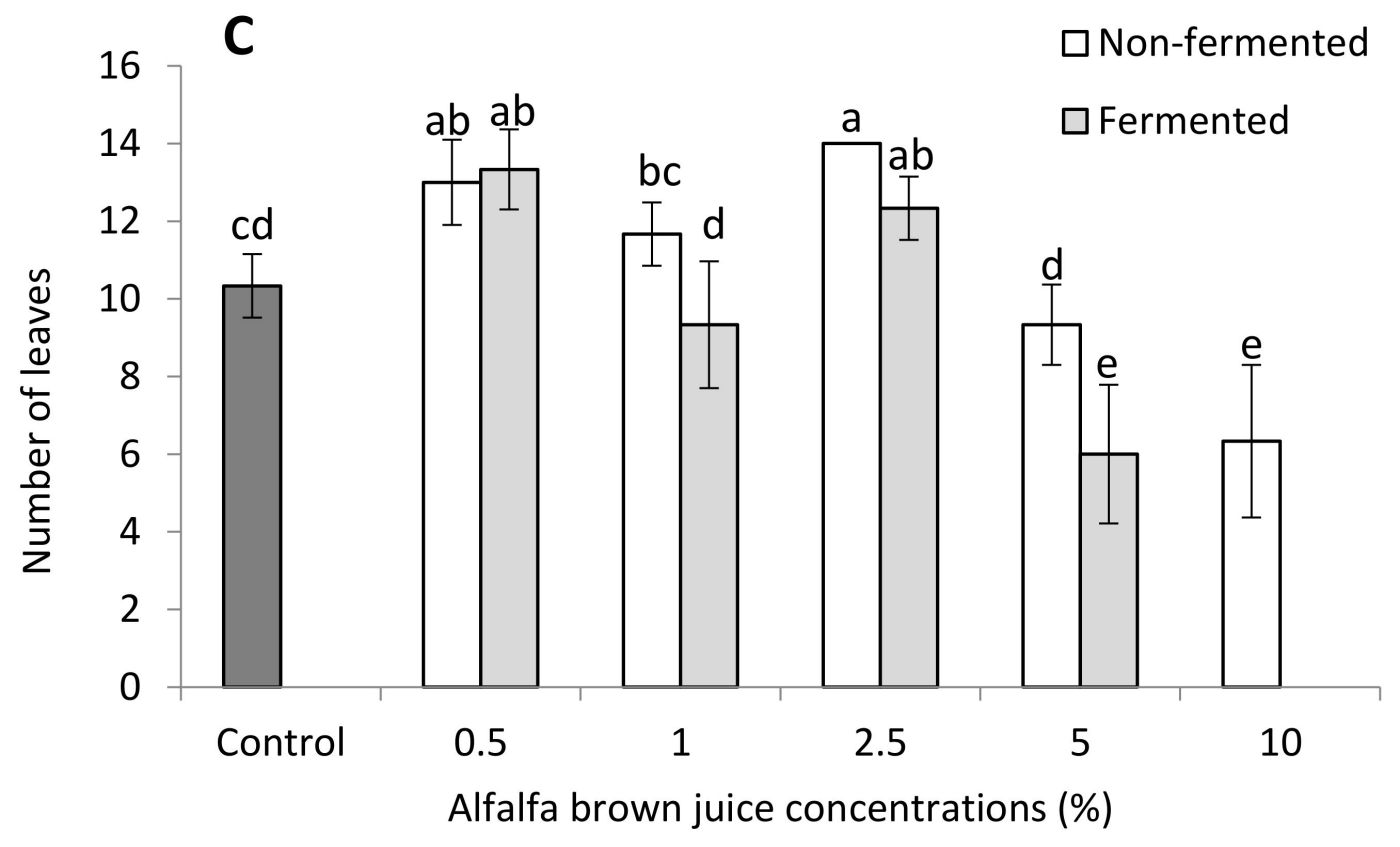
Effects of non-fermented and fermented alfalfa brown juice (BJ) on the (**A**) root length, (**B**) shoot length, and (**C**) number of leaves of French marigold plants (*n* = 6). Different letters on top of the bars indicate significant differences (*p* ≤ 0.05). Data are presented as mean ± standard deviation. Data are absent for 10% fermented BJ treatment due to the inhibitory effect of high BJ concentration; plants died.

**Figure 3:**
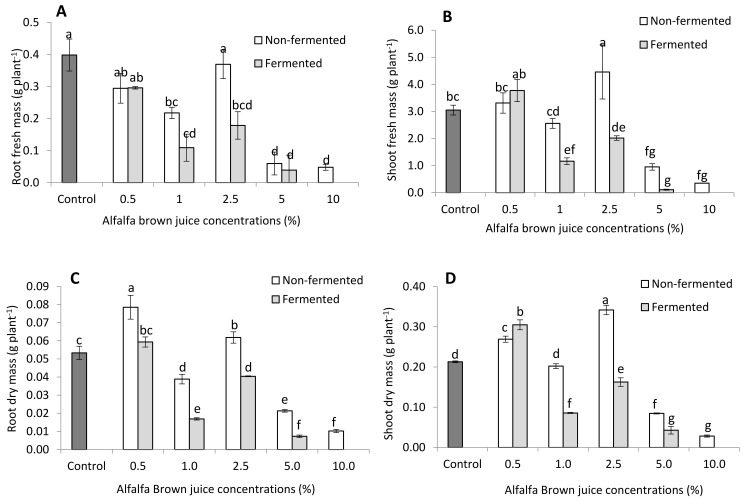
Effect of non-fermented or fermented brown juice (BJ) on the (**A**) root fresh mass, (**B**) shoot fresh mass, (**C**) root dry mass, and (**D**) shoot dry mass of French marigold plants (*n* = 3). Different letters on the top of the bars indicate significant differences (*p* ≤ 0.05). Data are presented as mean ± standard deviation. Data are absent for 10% fermented BJ treatment due to the inhibitory effect of high BJ concentration; plants died.

The plant biometrics results for French marigold plants treated with different doses of non-fermented and fermented BJ are presented in Figure 2 and Figure 3. The lowest BJ concentration (0.5%) enhanced plant growth, resulting in a deeper root system for treatments with both fermented (6.55 cm) and non-fermented (6.75 cm) BJ than in the control plants (6.07 cm). The deepest root system (7.87 cm) was measured in plants which received 2.5% of non-fermented BJ. Non-fermented BJ induced a greater growth of root than fermented BJ for all the treatments except 0.5% (Figure 2A). Similarly, plants treated with 0.5% of non-fermented and fermented BJ showed taller shoots of 8.23 and 9.50 cm, respectively, compared to the control plants (8.18 cm; Figure 2B). Moreover, the treatment of 2.5% non-fermented BJ had a shoot length of 9.22 cm, which was higher than that of the control plants. Plants treated with the lowest BJ concentration (0.5%) possessed a higher number of leaves than the control. Treatments of 1% of non-fermented BJ and 2.5% of fermented BJ also had higher numbers of leaves than the control (Figure 2C). However, the highest number of leaves (13.3) was measured for plants sprayed with 0.5% of fermented BJ.

All plants treated with non-fermented or fermented BJ had lower root fresh mass than the control plants. The results also showed that the fermented BJ reduced more of the root fresh mass than the non-fermented BJ, regardless of the applied concentration (Figure 3A). The control plants exhibited 0.40 g plant^−1^ root fresh mass, while the highest root fresh mass measured for the fermented and non-fermented BJ was 0.30 and 0.37 g plant^−1^, at concentrations of 0.5% and 2.5%, respectively. In contrast to root fresh mass, the lowest applied concentration of both non-fermented and fermented BJ led to higher shoot fresh mass compared to the control plants. At 0.5% of non-fermented and fermented BJ, shoot fresh masses of 3.31 and 3.77 g plant^−1^, respectively, were measured, while the control plants possessed a shoot fresh mass of 3.05 g plant^−1^ (Figure 3B). Nevertheless, the highest shoot fresh mass (4.46 g plant^−1^) corresponded to plants which received 2.5% of non-fermented BJ. Plants treated with non-fermented BJ displayed higher shoot fresh mass, regardless of the applied concentration, compared to plants treated with fermented BJ. Plants sprayed with 0.5% fermented and non-fermented BJ had a higher root dry mass than the control. For instance, root dry mass at 0.5% fermented and non-fermented BJ was 0.06 and 0.08 g plant^−1^, respectively, whereas the control plants had a root dry mass of 0.05 g plant^−1^ (Figure 3C). Shoot dry mass showed the same response to BJ as root dry mass where plants treated with 0.5% of non-fermented and fermented BJ had shoot dry mass of 0.27 and 0.31 g plant^−1^, respectively, which is higher than that of the control plants (0.21 g plant^−1^). The plants treated 2.5% of non-fermented BJ had the highest shoot dry mass of 0.34 g plant^−1^ (Figure 3D).

#### 2.2.2. Antioxidant Capacity of French Marigold Plants in the Presence of BJ

**Figure 4:**
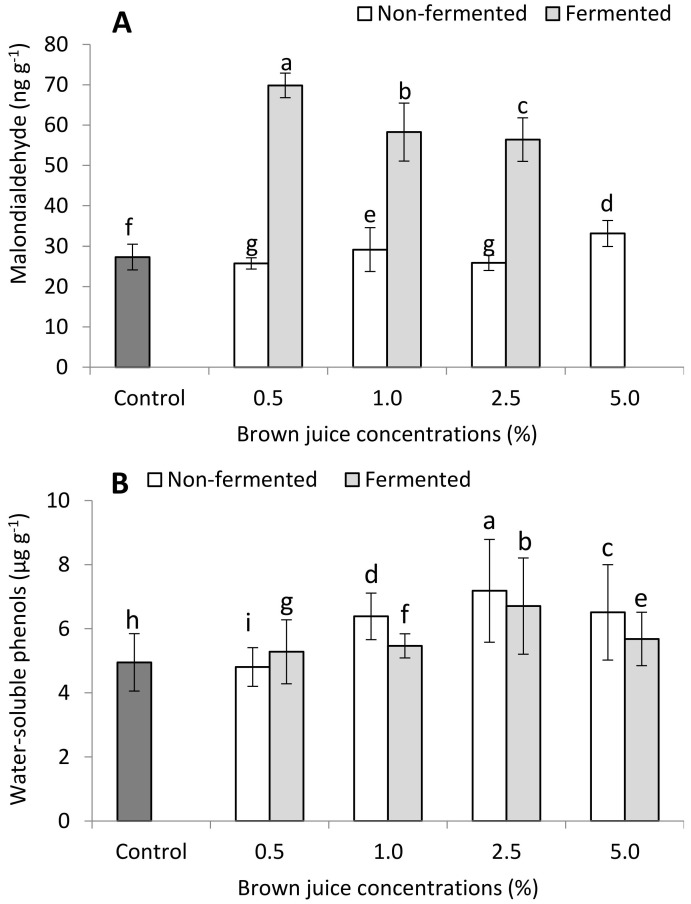

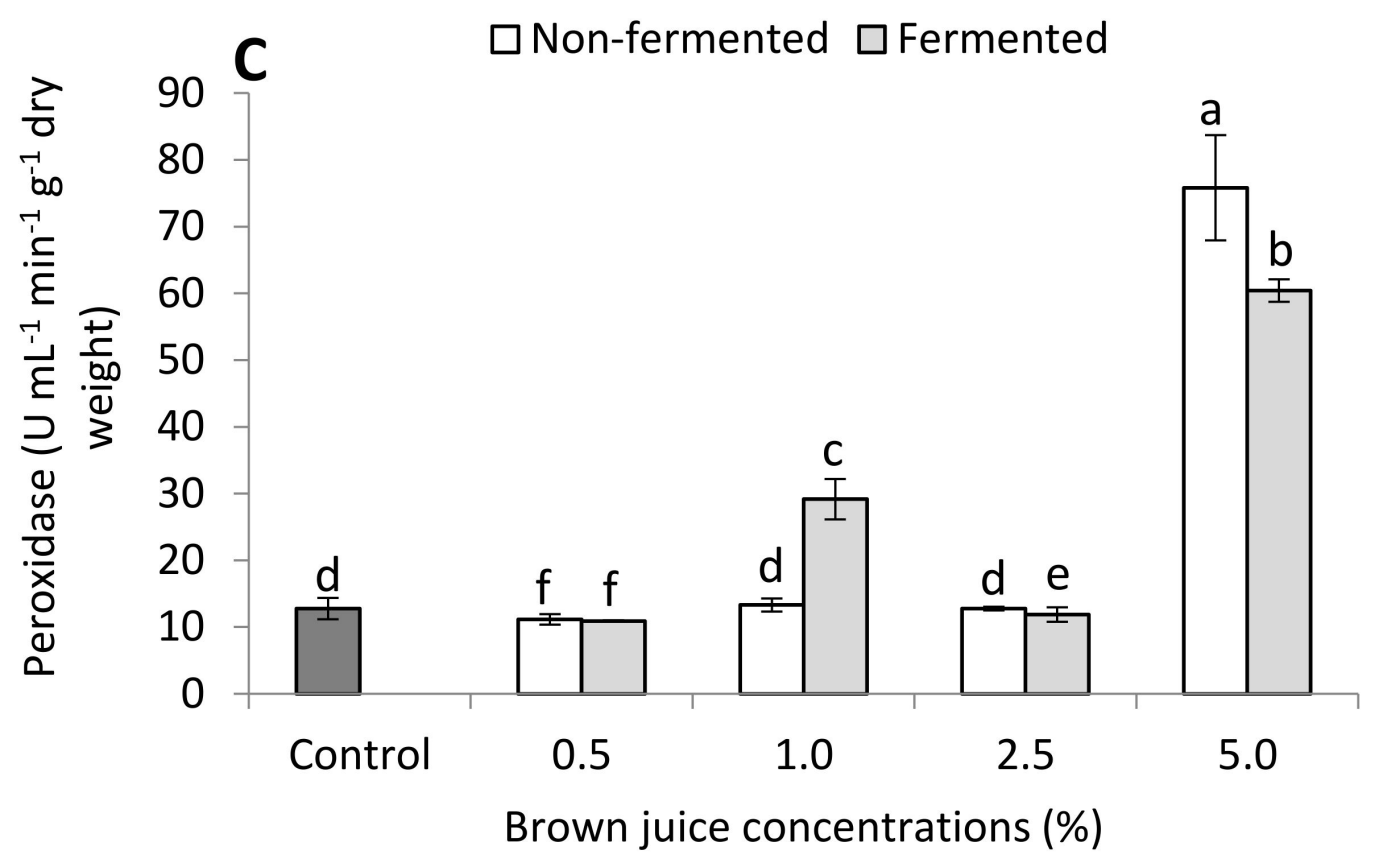
Effect of non-fermented and fermented BJ on the (**A**) malondialdehyde content, (**B**) water-soluble phenol content, and (**C**) peroxidase activity of French marigold plants (*n* = 10). Different letters on the top of the bars indicate significant differences (*p* ≤ 0.05). Data are presented as mean ± standard deviation. Data are absent for 5% (Figure 4A) fermented BJ treatment due to the inhibitory effect of high BJ concentration; plants died.

Figure 4 displays the results of some physiological indicators of the French marigold plants sprayed with fermented and non-fermented BJ. The plants treated with 5% fermented BJ showed poor growth, which is the cause of the missing data. All the concentrations of the fermented BJ showed higher MDA content than the control plants and the non-fermented BJ. The MDA content of plants which received the fermented BJ decreased gradually when the concentration of the applied fermented BJ was increased (Figure 4A). Plants treated with concentrations of 0.5% and 2.5% of non-fermented BJ displayed lower MDA content than the control plants. The highest MDA content (69.8 ng g^−1^) was measured for plants exposed to 0.5% of fermented BJ, while the control plants had a MDA content of 27.3 ng g^−1^ (Figure 4A). Except for the treatment of 0.5% non-fermented BJ, all of the treatments of fermented and non-fermented BJ had a higher content of water-soluble phenol compared to the control plants. Moreover, the water-soluble phenol content increased when the BJ content was increased up to 2.5%, and then decreased upon increasing BJ concentration to 5%, regardless of BJ type (Figure 4B). Non-fermented BJ showed higher water-soluble phenol content in all treatments except the 0.5% treatment. The control plants had a water-soluble phenol content of 4.95 mg g^−1^, while plants treated with 0.5% non-fermented BJ had a water-soluble phenol content of 4.80 mg g^−1^. However, the treatments of 0.5% and 2.5% fermented and non-fermented BJ exhibited lower POD activity (U mL^−1^ min^−1^ g^−1^ dry weight) than the control plants. Increasing the applied concentration of fermented and non-fermented BJ gradually increased the activity of POD. However, unexpected higher activity of POD was measured for the treatment of 5% fermented BJ (Figure 4C).

#### 2.2.3. Photosynthetic Pigments of French Marigold Plants in the Presence of BJ

**Figure 5:**
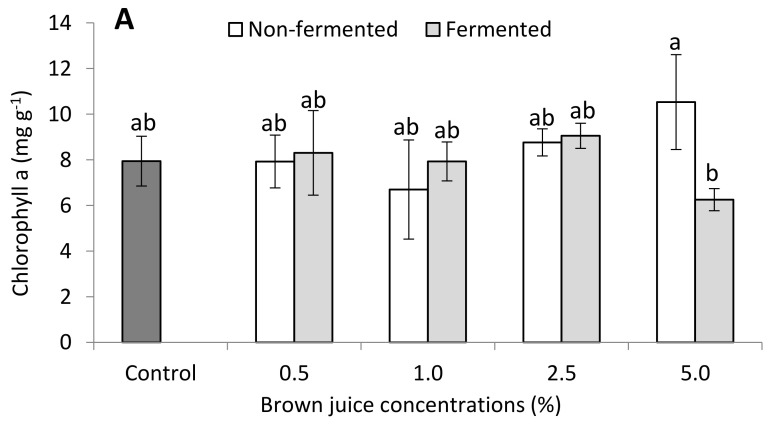

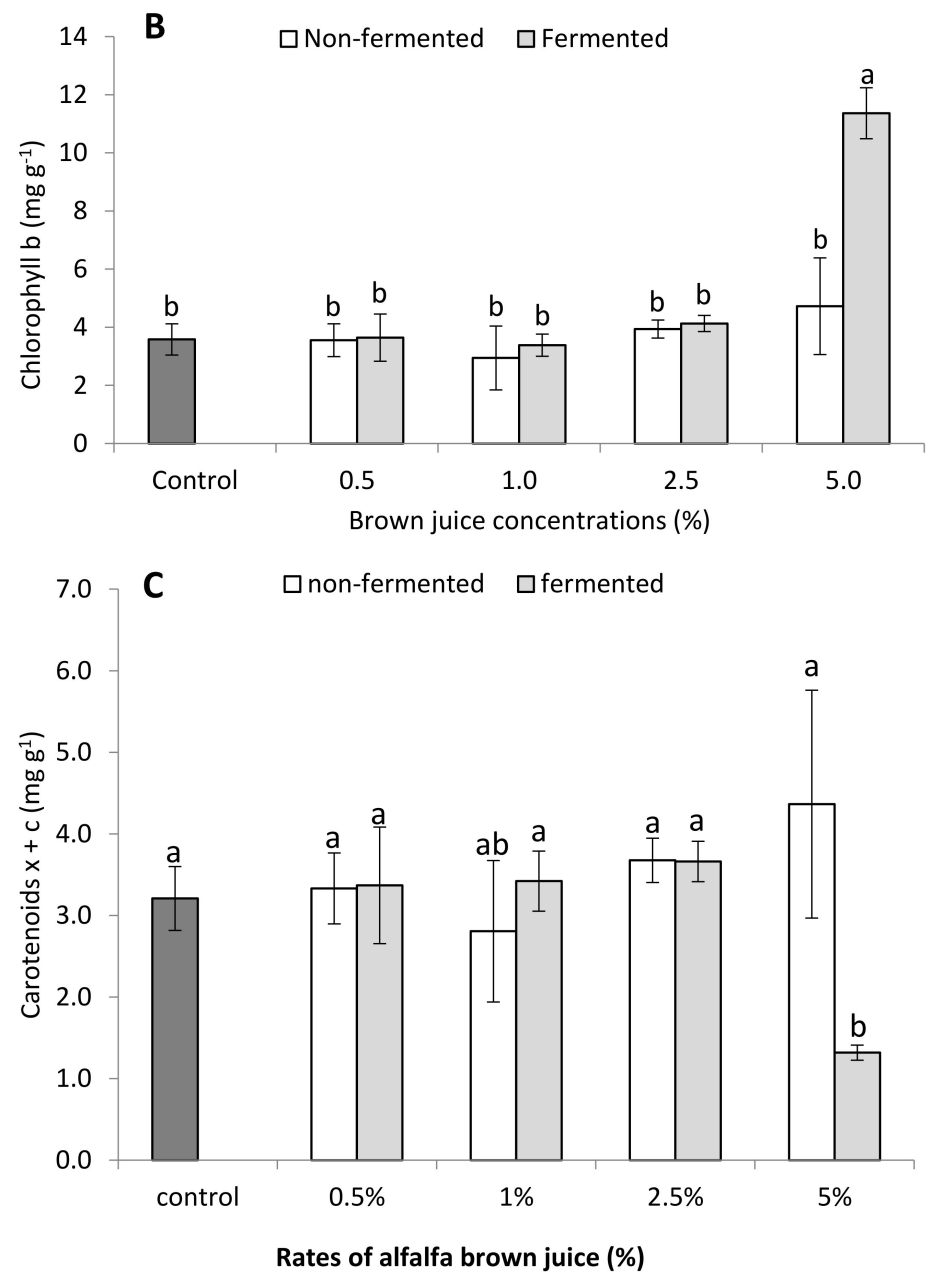
Effects of non-fermented and fermented alfalfa brown juice (BJ) on the (**A**) chlorophyll a, (**B**) chlorophyll b, and (**C**) (carotenoids (mg g^−1^) content of French marigold plants (*n* = 10). Different letters on the top of the bars indicate significant differences (*p* ≤ 0.05). Data are presented as mean ± standard deviation.

The results of the photosynthetic pigments, i.e., chl a, chl b, and carotenoids, after treating the French marigold plants with fermented and non-fermented BJ are presented in Figure 5. The plants treated with fermented BJ had higher values for all the pigments compared to control and non-fermented BJ treatments, except for the treatments of 5% for chl a and carotenoids (Figure 5). The plants treated with 0.5% and 2.5% fermented BJ had a higher chl a content than those in the control and non-fermented BJ treatments. However, the highest chl a content (10.53) corresponded to 5% non-fermented BJ. Applying BJ at concentrations of 2.5% resulted in the second highest chl a content after 5% of non-fermented BJ (Figure 5A). Similar results were reported for the chl b content. Except for the treatments of 1% fermented and non-fermented BJ, plants in all treatments exhibited higher chl b content than the control plants. Increasing the BJ concentration resulted in an increase in chl b content (Figure 5B). The positive effect of fermented BJ on photosynthetic pigments extended to substantially affect the carotenoid contents of the treated plants. The carotenoid content increased gradually as the concentration of fermented BJ and non-fermented BJ increased, except in concentrations of 1% of non-fermented BJ, which recorded lower values than the control and fermented BJ treatments (Figure 5C). The differences of concentration of non-fermented BJ appeared to be insignificant for all three parameters.

### 2.3. Effect of Non-Fermented and Fermented BJ on the Anatomical Features of French Marigold

**Figure 6:**
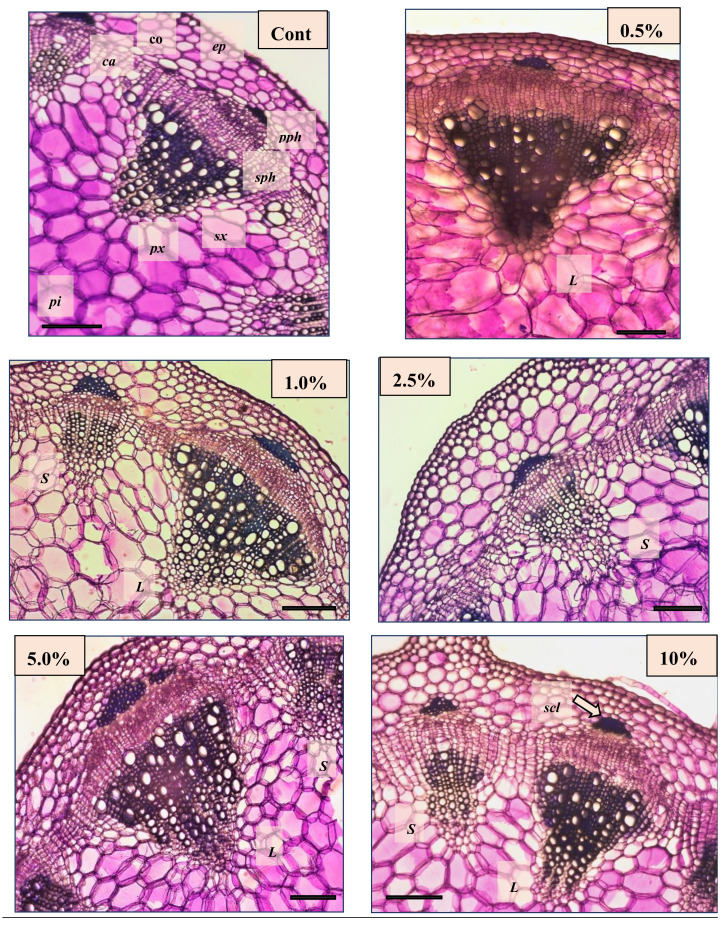
Anatomical sections of a French marigold stem after spraying with different rates of fermented alfalfa brown juice (BJ) (i.e., Control, 0.5%, 1%, 2.5%, 5%, and 10 %). L large vascular bundle, S small vascular bundle, ep epidermis, co cortex, pi pith, pph primary phloem, sph secondary phloem, ca cambium, px primary xylem, sx secondary xylem, and scl sclerenchyma. The scale bar is 200 µm.

The tissue structure of the French marigold showed the tissue structure of a typical dicotyledonous plant after thickening. It was characterized by an epidermis (with cuticle), a thin cortex of parenchymatic and collenchyma cells (4–6 rows), and a pith containing secondary vascular tissues. There were no fundamental differences in the anatomical features of the plants from the different treatments, but they differed in their proportion and extension of tissues. There were four typical longitudinal ribs running along the stem of the plants from each treatment. In the ribs, the angular collenchyma consisted of more cell rows than the other parts of the stem did. At low BJ concentrations (control, 0.5%, and 1%), an average of 3–4 rows of collenchyma was found in the ribs, in contrast to at higher concentrations (2.5%, 5%, 10%), at which 5–7 collenchyma cell rows stiffened the stem. Moreover, treatment with higher concentrations of BJ resulted in the development of a collenchyma in the primary phloem. The French marigold shows a thickening of the helianthus type. The essence of this type is that the interfascicular cambium is formed, creating new, smaller-sized vascular bundles that contain only secondary tissue (secondary xylem and phloem). For all treatments, six large and six small bundles were identified (alternately), except for the 5% treatment, in which seven small bundles were observed (Figure 6).

Increasing the concentration of BJ in each treatment significantly increased the parameters describing stem strength. The development and secondary thickening of the control plants lagged far behind the plants treated with BJ. For all parameters, the 5% treatment was the most effective (except for the secondary xylem of large bundles) and the differences were always statistically significant. Although the 10% treatment caused only a small increase in the proportion of secondary tissues and sclerenchyma compared to the control, these differences were statistically significant. Additionally, a 2.5% treatment was recommended to stimulate thickening (Table 1).

## 3. Discussion

Recently, there has been interest to reduce dependency on inorganic fertilizers and plant growth stimulants with an inorganic origin in ornamental plants. The use of bio-origin nutrients and growth promoters supports the organic farming approach and the sustainability concept. In the present study, we investigated the effect of an organic-derived biostimulant (i.e., BJ) on the seed germination process and plant development of a well-known ornamental plant, i.e., the French marigold. Seeds and seedlings were treated with different concentrations (i.e., (control) 0%, 0.5%, 1.0%, 2.5%, 5.0%, and 10%) of non-fermented and lacto-fermented BJ. The BJ is more than a by-product of the LPC process. In our previous related studies, we showed the extent to which BJ is rich in several macro- and microelements, beside many other compounds with growth-promoting effects, such as phenols, sugars, and organic acids, in addition to lactic acid bacteria in the case of fermented BJ, which has an additional value [3,8]. Previous studies examined the seed germination of *Tagetes* species in the presence of poultry manure [20,22] and pharmaceutical residues [23], but plant-derived biofertilizers were not tested in scientific detail. In the present study, fermented BJ, in general, showed higher values than non-fermented BJ for all the measured germination indices. Our findings also revealed that 0.5% and 1.0% fermented or non-fermented BJ, respectively, were the most effective treatments. However, 0.5% fermented BJ exhibited a higher promoting effect compared to 1.0% non-fermented BJ. The 0.5% fermented BJ resulted in increases of 9.6%, 11.2%, 10.9%, and 41.7% of FGP, GRI, GI, and VI-I, respectively. Similar results were obtained for the seed germination of the French marigold by Majkowska-Gadomska et al. [28]. They cited that *Trichoderma* spp. and Goёmar Goteo significantly enhanced seed germination of the French marigold compared to the China aster (*Callistephus chinensis* (L.) Nees), scarlet sage (*Salvia splendens* Sellow ex Roemer and J.A. Schultes), and common zinnia (*Zinnia elegans* Jacq.).

Higher concentrations, i.e, above 2.5%, of fermented or non-fermented BJ significantly reduced all the germination indices. Seeds exposed to 10% fermented BJ were almost unable to germinate. This could be ascribed to the lower pH (5.09) of 10% fermented BJ compared to 5.23 for the same concentration of non-fermented BJ (Table 2). Furthermore, soaking seeds in BJ solutions for two hours showed higher absorption of the BJ solution at the concentration of 10% fermented BJ, compared to lower BJ concentrations. This could introduce an acceptable explanation for the inability of seeds left to germinate on 10% fermented BJ, which had the lowest pH. However, a lower amount of non-fermented BJ solution was absorbed by the seeds. The penetration of a more acidic solution into the seeds could damage seed vitality, causing a reduction in germination energy. Additionally, low pH negatively affects seed germination [28]. Turner et al. [29] mentioned that no seed germination occurred below a pH of 4 and that a pH lower than 5.5 substantially diminished the development of seedlings, recording lower shoot and root biomass and lengths. The stimulating effect of BJ on plant growth has still not been completely revealed. The optimal concentration of minerals, bioactive molecules, composition of plant growth-promoting bacteria, and phytohormones probably play a role [3,7,8]. Additionally, seeds treated with high amounts of BJ have been infected by pathogens. Apparently, BJ is also an ideal medium for pathogenic fungi, such as *Fusarium* spp., *Phytophtora* spp., *Rhizoctonia solani*, and bacteria (e.g., *Pseudomonas* spp.). The slow germination and high concentration of nutrients could be the major cause of this abundance of pathogenic microorganisms [30]. Poor germination could also be a result of osmotic stress [31]. The BJ contains minerals and sugars, which makes it harder to infiltrate the seeds. However, concentrations of BJ below 10% were reported to induce seed germination of several plants, such as the growth of cowpeas, mung beans, and groundnuts [32].

Across all the tested vegetative parameters, the 0.5% fermented and 2.5% non-fermented BJ were the most promising, resulting in the highest values of length of root and shoot, fresh and dry masses of root and shoot, and number of leaves. Increasing the concentrations of either fermented or non-fermented BJ resulted in a significant decrease in the values of plant biometrics below the levels of the control plants. In our previous research, 0.5% and 1.0% fermented BJ were reported to induce the development of *Celosia* plants compared to control plants and higher concentrations of fermented BJ. The synergetic effect of fermented BJ could be due to its high content of macro- and microelements [3]. In addition, the application of non-fermented BJ annually at a rate of 1.25 cm was reported to significantly enhance the growth and productivity of alfalfa, corn, and brome grass [33]. Higher rates were found to reduce the growth of the same crops. The reduction in plant growth and seed germination could be attributed to some toxic phytochemicals that are found in BJ, as reported previously by Pirie [34].

The photosynthetic pigment content is a direct indicator of plant fitness [35]. Increasing the concentration of the applied BJ solution gradually increased the chl a, b, and carotenoids content of the seedlings. Increasing the concentration of fermented BJ gradually increased the content of chl a, while non-fermented BJ had the highest chl a content (10.53 µg ml^−1^) in plants that received 10% BJ. Similar results were noticed for the chl b and carotenoid contents in plants treated with both fermented and non-fermented BJ.

Antioxidant capacity is of substantial importance. In plant growth stimulation, antioxidant molecules could serve as key contributors in the protective mechanisms against biotic and abiotic stress [16,36,37]. The low rate of fermented BJ (0.5%) showed the lowest content of water-soluble phenols, and POD activity, while the same concentration of fermented BJ resulted in slightly higher values of antioxidant indicators. Interestingly, plants treated with fermented BJ had higher MDA contents than the control plants and non-fermented BJ, regardless of the applied dose of BJ. These results prove the potential to use BJ as a growth biostimulator in its fermented or non-fermented form, especially at low concentrations, below 0.5%.

Another possible reason for the reduction in plant growth could be the high osmotic pressure of the more concentrated BJ, and the high EC value associated with the low pH. Increasing the concentrations of BJ gradually increased EC and decreased pH. However, fermented BJ showed a lower increase in EC and higher decrease in pH compared to non-fermented BJ (Table 2). Plants grown on growth medium at a low pH suffer from a reduction in the rate of the photosynthesis process due to the reduction in stomatal conductance caused by low pH [38]. Moreover, when citrus is grown below a pH of 4, its growth reduced [39], while there is better plant development at higher pH values. The negative impact of low pH on plant development could be attributed to ion toxicity due to high H^+^ concentration in low pH, causing the burning of leaves. Data outlined in the present study prove the efficiency of alfalfa BJ as a plant growth biostimulator regardless of its lacto-fermentation. However, the fermentation of BJ using lactic acid bacteria is needed to store the fresh BJ for longer periods. Additionally, due to its richness in macro- and microelements and organic acids, BJ can be recommended for use on sandy and alkaline soils as conditioner and fertilizer.

## 4. Materials and Methods

### 4.1. Source of Brown Juice (BJ) and Plant Materials

Fresh alfalfa BJ was obtained from the Proteomill Green Protein Biorefinery Factory (Tedej Ltd., Hajdúnánás, Hungary) as described by Bákonyi et al. [3]. The seeds of French marigold (*Tagetes Patula* L. ‘Csemő’) were obtained from the National Agricultural Research and Innovation Center (NARIC, Budapest, Hungary).

### 4.2. Seed Germination Experiment

We conducted a germination test to examine how different fermented and non-fermented BJ doses affect the seed germination of French marigold. The germination test was conducted in Petri dishes with a 9 cm diameter with two layers of grade 3 hw qualitative filter paper (Ahlstrom-Munksjö, Munktell Filter AB, Sweden) without light in a Phytotron (HOTPACK Series 922, Philadelphia, PA, USA). The environmental conditions were controlled; the temperature was 19 °C and the relative humidity was set to 60%. Twenty-five seeds were used per Petri dish with four repetitions. Each Petri dish received 2 mL of 0.5%, 1.0%, 2.5%, 5.0%, or 10% of fermented or non-fermented BJ, and distilled water was used as a control, making a total of 11 treatments. We measured the pH and electrical conductivity (EC) values of the solutions used in the germination experiment (Table 2). Then, the seedlings were disposed of, and we used new plants in the other experiments.

The number of germinated seeds was counted daily, and seedlings with a 5 mm radicle were considered as a germinant. The mean germination time (MGT), final germination percentage (FGP), coefficient of velocity of germination (CVG), germination rate index (GRI), and germination rate (GI) were calculated following Alsaeedi et al. [40] and Abdul-Baki and Anderson [41].

### 4.3. Greenhouse Experiment Setup

A pot experiment was conducted under greenhouse conditions at the NARIC to assess the possible growth stimulation effect of fermented and non-fermented alfalfa BJ using the French marigold as a plant model. The experiment was conducted alongside our previously published works [3,8]. The experimental design was the Randomized Complete Block design (RCB) with 3 repeats. A polyethylene pot (7 × 7 × 8 cm) was filled with white peat for young plants (Klassman-Deilmann TS 3 FINE type, Geeste, Germany). The characteristics of growth medium are as follows: fine structure, pH (H_2_O) 6, N 140 mg L^−1^, P (P_2_O_5_) 100 mg L^−1^, K (K_2_O) 180 mg L^−1^, Mg 100 mg L^−1^, and S 150 mg L^−1^. The germinated seedlings were treated with fermented and non-fermented BJ at different rates (0.5%, 1.0%, 2.5%, 5.0% and 10%). Tap water was applied to the control seedlings. Samples were collected for the further biometric, physiological, and anatomical analyses. As we observed in the previous pot experiments [3,8], the 10% percent BJ treatment showed an inhibitory effect and resulted in reduced biomass, which was insufficient for further measurements, such as the physiological experiments.

#### 4.3.1. Determination of Peroxidase

Peroxidase (POD) activity in lyophilized roots, stems, and leaves of the French marigold plants was determined following Roxas et al. [42]. Briefly, 200 mg of plant tissues was macerated in 1 mL of phosphate buffer 0.01 M (pH 6.0). The homogenate was centrifuged at 13,000 rpm for 10 min to collect the supernatant. The total volume of the reaction mixture was 1760 µL, containing 1700 µL Na-acetate buffer (01 M pH 50), 30 µl 0.3% H_2_O_2_, 20 µL o-Dianisidine (10 mg mL^−1^), and 10 µL leaf extract. The supernatant was used to measure POD activity using an Ultrospec 2100 Pro UV/VIS spectrophotometer (Amersham BioSciences) at 440 nm for 1 min with 10 second intervals. The extinction coefficient of o-dianisidine is 11.3 mM^−1^ cm^−1^ at 440 nm. Calculation was carried out according to the following equation:

Calculation: U mL^−1^ = (Y × 11.3^−1^) × 1760 × 10^−1^

Y = you can represent the data in figures and superposing the trendline on the plots and y value; 11.3 = extinction coefficient of o-dianisidine; 1760 = total volume in the cuvette (µL); and 10 = sample volume in the cuvette (µL).

The unit of POD activity was defined with the increase of one unit of absorbance mL^−1^ min^−1^ g^−1^ of dry matter.

#### 4.3.2. Malondialdehyde and Water-Soluble Phenol Measurement

The malondialdehyde (MDA) content was determined from the shoot of French marigold plants using the method of Zang and Huang [43]. Briefly, 100 mg of lyophilized sample was homogenized in 1 mL 0.1% (*w*/*v*) TCA solution using a cold mortar and pestle. The homogenates were centrifuged at 10,000× *g* for 10 min. Then, 4 mL of 0.5% thiobarbituric acid (TBA) in a 20% TCA solution was added to 1 mL of supernatant and incubated at 96 °C for 30 min. Following this, the tubes were immediately cooled by transferring them into an ice bath. The absorbance of the supernatant was recorded at 532 nm. The standard curve was generated using MDA as a standard. The concentration of the MDA in the samples was calculated using the absorbance calibration curve.

The water-soluble phenol content in the shoot tissues of French marigolds was determined using Folin–Ciocalteu reagent, following the method of Box [44]. Tannic acid was used as the standard and the concentration of water-soluble phenol was expressed as tannic acid equivalents (mg TAE g^−1^ DW).

#### 4.3.3. Photosynthetic Pigment

The content of photosynthetic pigments of the French marigold leaves was measured spectrophotometrically following Moran and Porra [45] and Wellburn [46]. For sample preparation, a 0.05 g leaf disc was cut and the chlorophyll content was extracted using 5 mL *N*,*N*-dimethylformamide overnight. The absorbance was measured using a spectrophotometer (Amersham Biosciences, Ultrospec 2100 Pro UV/Visible) on 664 (chl a) and 647 nm (chl b), 480 nm (carotenoids) wavelengths, and from these data, the chl a, b, and carotenoid contents were calculated based on the following equations:

Chlorophyll a (mg g^−1^) = (11.65 a664 − 2.69 a647),

Chlorophyll b (mg g^−1^) = (20.81 a647 − 4.53 a664),

Total carotenoids (mg g^−1^) = ((1000 × a480 − 1.28 × chl a) − (56.7 × chl b)) × 100^−1^.

### 4.4. Plant Anatomy

The third internode from the base of each plant was used for analysis. Stem pieces were placed in a preservative solution and then incised using a blade. Cross-sections were stained with toluidine blue (2%). Plant anatomical features were examined using light microscopy (Zeiss Axioscope 2+; Zeiss International, Oberkochen, Germany), and digitized photographs were taken. Using measurement software (Scope Photo, Scopetek, München, Germany), we measured 45 data points per parameter for each treatment to use in analysis. We measured several different parameters, including the diameter of the stem, cortex, pith, thickness of the epidermis, and number and diameter of vascular bundles. Before microscopic observations, the plant parts were kept in a 1:1:1 ethanol:glycerol:water solution. This paper focuses on the parameters representing the thickness and strength of the stem. Therefore, the secondary xylem of different types of bundles (large and small) and sclerenchyma caps at the apex of the bundles are highlighted in the evaluation.

### 4.5. Statistical Analysis

We used SigmaPlot 12.0 and IBM SPSS Statistics 24 software to evaluate the results. The data showed homogeneity after being subjected to Shapiro–Wilk normality testing. Levene’s test for equality of variances was performed to analyze the data. We found an interaction of factors. Therefore, we used the combination of the factors (fermentation, concentration) to conduct a one-way ANOVA. Groups were compared using the Holm–Sidak method [47] at *p* ≤ 0.05. We used the control as a pseudo treatment.

In the case of Figure 2 and Figure 3 ‘5% fermented’ and Figure 4A ‘10% fermented’ BJ treatment, data are absent due to the inhibitory effect of high BJ concentration, i.e., the plants died.

## 5. Conclusions

In this study, we examined the effect of regular alfalfa BJ applications in the early developmental stages of *Tagetes* plants. Our findings agreed with findings from previous experiments; higher concentrations (>2.5%) of BJ showed an inhibitory effect during the later stages of ontogenesis in plants. In addition, BJ was found to improve various physiological parameters and induce morphological changes, which could be beneficial, particularly for ornamental purposes. It could be concluded that the appropriate concentration (mainly 0.5% to 2.5%) of alfalfa BJ had a positive effect on the early-stage development of *Tagetes* plants and, together with our previous results for sweet basil and plumed cockscomb, we suggested that alfalfa BJ is a plant biostimulant.

As the average population becomes more and more environmentally conscious, the popularity of natural products increases. Edible flowers, such as *Tagetes*, are currently trending, which raises awareness towards clean, pesticide-free plant parts. Using appropriate amounts of alfalfa BJ on plants could contribute to the reasonable application of this valuable by-product and reduce negative environmental impacts, such as releasing BJ from protein mill factories directly to fields, as well as promoting environmentally friendly pesticide-free edible flower production.

Besides being a potential plant conditioner, fermented alfalfa BJ could be exploited as a biofertilizer for soil in low concentrations. The use of biofertilizers allows us to reduce the use of chemical fertilizers and pesticides, which results in less severe environmental impacts, and reduced costs in general. Further investigations should be conducted to unveil the exact mechanisms in the rhizosphere behind the effects of this complex plant extract. Currently, fermented BJ can be implemented by organic and conventional farmers to meet the demand of consumers and the expectations of legislators.

## Figures and Tables

**Table 1:** Impact of different concentrations of fermented alfalfa brown juice (BJ) on some characteristics of the stem tissue of French marigold (µm) (mean ± standard deviation, *n* = 45).

	Diameter (Stem)	Large Vascular Bundles			Small Vascular Bundles		
		Diameter (Bundle)	Secondary Xylem	Sclerenchyma	Diameter (Bundle)	Secondary Xylem	SCLERENCHYMA
**Cont**	3172.64 ± 109.84c	539.11 ± 63.36c	191.33 ± 44.91d	26.97 ± 8.64c	327.56 ± 40.57d	189.73 ± 38.57d	34.42 ± 9.02c
**0.5%**	3537.60 ± 235.97b	603.41 ± 84.27b	274.23 ± 67.53b	44.08 ± 11.42b	414.69 ± 66.45ab	271.86 ± 62.56b	56.53 ± 17.06b
**1%**	3669.29 ± 257.50ab	621.45 ± 32.82b	288.66 ± 41.93b	42.05 ± 12.51b	347.24 ± 34.27bd	256.10 ± 42.50b	54.80 ± 12.54b
**2.5%**	3751.34 ± 174.37a	660.55 ± 59.15a	317.23 ± 60.16a	39.86 ± 11.41b	392.04 ± 44.40b	277.75 ± 45.62b	50.81 ± 14.04b
**5%**	3802.14 ± 141.03a	676.12 ± 63.15a	285.16 ± 49.41b	54.61 ± 14.83a	431.06 ± 71.77a	312.08 ± 66.55a	60.27 ± 13.25a
**10%**	3504.17 ± 186.73b	610.87 ± 75.64b	240.98 ± 48.65c	45.13 ± 11.50b	361.14 ± 50.91c	238.52 ± 47.36c	48.85 ± 11.54b

Different letters in each column indicate statistically significant differences (*p* < 0.05).

**Table 2:** pH and electrical conductivity (EC) values of fermented and non-fermented brown juice (BJ) solutions.

BJ Rates (%)	pH		EC (dS m^−1^)		Absorbed Solution (g 10^−1^ seeds 2 h^−1^)	
	Non-Fermented	Fermented	Non-Fermented	Fermented	Non-Fermented	Fermented
Distilled water	6.91		0.0		0.032	
0.5	5.68	5.51	0.19	0.16	0.031	0.028
1.0	5.61	5.39	0.35	0.28	0.029	0.030
2.5	5.43	5.28	0.81	0.64	0.025	0.027
5.0	5.33	5.19	1.43	1.20	0.024	0.032
10.0	5.24	5.09	2.54	2.11	0.026	0.032
